# Characteristics of Peripheral Blood Lymphocyte Populations in Patients with Locally Advanced Unresectable Non-Small Cell Lung Cancer

**DOI:** 10.3390/cancers17213504

**Published:** 2025-10-30

**Authors:** Lyudmila Grivtsova, Anastasia Glukhareva, Anzhelika Melnikova, Tatiana Mushkarina, Grigoriy Afonin, Anna Efremova, Sergey Ivanov, Dmitry Goldshtein, Andrey Kaprin

**Affiliations:** 1 A. Tsyb Medical Radiological Research Center (MRRC)—The Branch of the FSBI “National Medical Research Radiological Centre” (NMRRC) of the Ministry of Health of the Russian Federation, 4 Koroleva St., Obninsk 249036, Russia; grivtsova@mail.ru (L.G.); angelik_melnikova@mail.ru (A.M.); jeweltany@gmail.com (T.M.); dr.g.afonin@mail.ru (G.A.); ivanov.obninsk@mail.ru (S.I.); 2 Research Centre for Medical Genetics, 1 Moskvorechye St., Moscow 115522, Russia; anna.efremova@med-gen.ru (A.E.); dvgoldshtein@med-gen.ru (D.G.); 3 Research Institute of Molecular and Cellular Medicine and Biochemistry Department, Peoples’ Friendship University of Russia, 6 Miklukho-Maklaya St., Moscow 117198, Russia; mail@nmicr.ru; 4 FSBI “National Medical Research Radiological Centre” of the Ministry of Health of the Russian Federation, 10 Marshal Zhukov St., Obninsk 249031, Russia; 5 P. Hertsen Moscow Oncology Research Institute (MORI)—The Branch of the FSBI “National Medical Research Radiological Centre” (NMRRC) of the Ministry of Health of the Russian Federation, 3 2nd Botkinsky proezd, Moscow 125284, Russia

**Keywords:** non-small cell lung carcinoma (NSCLC), peripheral blood lymphocyte subpopulations, flow cytometry

## Abstract

**Simple Summary:**

Lung cancer was reportedly the most commonly diagnosed cancer in both men and women in 2024, and approximately 80 to 90% of all cases were non-small cell lung cancer (or carcinoma, NSCLC). The aim of this study was to investigate characteristics of blood immune cell population in patients primarily diagnosed with stage IIIB, IIIC NSCLC in order to identify potential signs of secondary immunodeficiency. Quantitative and qualitative changes at the level of individual immunocompetent cell populations were detected in 80 of primary NSCLC patients. The identified multidirectional changes in immunocompetent cells (T-, B-, NK-, and NKT cells) confirm the existence of secondary immunodeficiency in this group of patients.

**Abstract:**

Background: The main type of treatment of unresectable NSCLC is chemoradiotherapy, which has a relatively high toxicity. One option allowing to reduce the toxicity of this approach may be immunocorrective therapy. The appointment of this type of treatment should be warranted in terms of patient’s immune system response. This confirms the importance of verifying systemic immune disorders in primary patients with NSCLC. Goal: To assess the features of the population of immune cells in peripheral blood in patients with stage IIIB, IIIC primary NSCLC and to identify any signs of secondary immunodeficiency in this cohort. Methods: We analyzed the frequencies of circulating T cells (CD3+, CD4+, CD8+), B-cells (CD19+), NK-cells (CD3-CD16+CD56+ cell), and NKT-cells (CD3+CD56+ cells) within CD45+ cells (lymphocytes) in 80 patients with stage IIIB-IIIC NSCLC, and in 40 healthy controls using eight-color flow cytometry. Results: In patients with stages IIIB-IIIC primary NSCLC, changes within immunocompetent blood cells were found. Moreover, it was unveiled that quantitative changes affected all major immunocompetent cells. A decrease in the proportion of CD4+ T cells and B lymphocytes and an increase in the number of NK and NKT cells were found. Also, an increase in the number of double-positive CD4+CD8+ T cells was revealed, as well as a significant increase in the proportion of B1a (CD5+CD19+) cells among B lymphocytes (qualitative disorders). Conclusion: The revealed multidirectional changes among immunocompetent peripheral blood cells in patients with locally advanced NSCLC (stages IIIB-IIIC) can be beyond doubt considered as signs of systemic immune disorders in this cohort (secondary immunodeficiency).

## 1. Introduction

Lung cancer is still a significant health problem, ranking high in terms of morbidity and mortality worldwide, including the Russian Federation. It is the second most commonly diagnosed type of cancer among all malignant tumors. The implementation of active screening programs has led to an increase in the detection of early-stage lung cancer by 3.9% per year from 2013 to 2017. However, a majority of patients (approximately up to 70%) are diagnosed with advanced stage III or IV of the disease at the time of detection [1,2,3]. In the United States, Europe, and Russia, approximately 80–90% of lung cancer cases are non-small-cell lung carcinoma (NSCLC). The major histological subtypes of NSCLC include adenocarcinoma (AC), squamous cell carcinoma (SCC) and large cell carcinoma, while AC and SCC are the most common [4,5,6]. Over the past 20 years, treatment strategies for NSCLC have significantly improved, and targeted therapy is becoming cornestone. Targeted therapy in selected cohorts of patients (EGFR, ALK, ROS1, and NTRK mutations) and immunotherapy with checkpoint inhibitors leads to improved oncological outcomes [7,8]. However, only 15–20% of patients have indications for this type of therapy, and the effectiveness is often limited due to the development of tumor resistance to treatment [9].

For patients with locally advanced unresectable NSCLC, chemoradiotherapy is the primary treatment option. Previous studies have shown that concurrent chemo-radiotherapy (CCRT) is more effective than sequential [10]. However, this treatment has limitations, and one of the major challenges is its high toxicity. The toxicity prevention and treatment of complications of chemoradiotherapy do not provide 100% effectiveness [11]. Therefore, researchers are constantly exploring new approaches to reduce the toxicity of chemoradiotherapy and to minimize the risk of complications during both early and late stages of treatment. Among the possible therapeutic strategies for patients in this cohort, immunotherapeutic methods may also be considered [12]. Nevertheless, in order to justify the appointment, it is necessary to confirm the presence of a secondary immunodeficiency in this group of cancer patients.

The aim of our study is to identify possible immunological signs of secondary immunodeficiency based on the assessment of blood lymphocyte populations in patients with locally advanced inoperable stage IIIB, IIIC NSCLC before the therapy. A deeper understanding of this baseline immune status is crucial for optimizing immunology-based clinical strategies, namely, immune checkpoint inhibitors (ICIs) and adoptive cell therapy [13].

CD8+ cytotoxic T cells, CD4+ T-helper cells and CD3-CD16+CD56+ natural killers (NK cells) are key players in the antitumor immune response, and individual populations of CD4+ lymphocytes have immunosuppressive properties and can contribute to the depletion of cytotoxic NK and T cells [14,15].

Circulating lymphocytes, as possible biomarkers of prognosis, have attracted considerable attention from researchers in recent years, in particular the association of separate lymphocyte subset with the effectiveness of therapy in lung cancer [16]. In a recent study of a small patient cohort (*n* = 10), researchers used single-cell RNA sequencing (scRNA-seq) and antibody labeling (CITE-seq) in order to identify distinct subsets of circulating NK cells. The analysis revealed differences in cellular composition, gene expression, and signaling pathways associated with the time point of sampling and the NSCLC subtype [17].

Immunophenotypic features of circulating CD3+ and CD8+ T cells, in particular, decreased expression of activation markers (CD38); apoptosis molecules (CD95 phenotype) and perforin were reportedly associated with advanced lung cancer [18].

The identification of changes in peripheral blood lymphocytes composition can become the basis for the development of concomitant immunotherapy methods in this cohort of patients. This approach will allow to avoid increasing the intervals between courses of specific therapy, reducing the doses of chemotherapy drugs, which will undoubtedly have a positive effect on oncological outcomes (progression-free survival and overall survival).

## 2. Materials and Methods

This work was conducted as part of the scientific research project “Randomized study on the impact of concurrent immunotherapy on immediate outcomes of chemo-radiotherapy for patients with locally advanced, unresectable non-small cell lung cancer”, which was approved by the Local Ethics Committee of the National Medical Research Center for Radiology of the Ministry of Health of the Russian Federation (protocol No 13, dated 23 June 2022).

The study included analysis of blood samples from 80 patients with locally advanced unresectable NSCLC (stages IIIB-IIIC) before treatment and blood samples from 40 healthy volunteers (donors). The general characteristics of the patient group are presented in Table 1.

All patients included in the study did not have any contraindications for simultaneous chemo-radiotherapy, such as severe somatic pathology, an extremely severe condition (ECOG 4), active infectious disease, previous myocardial infarction, acute cerebrovascular accident (stroke) within the last 6 months, acute deep vein thrombosis (up to 5–7 days), tumor growth into large vessels or their disintegration, a threat of bleeding from the irradiated area, or cancerous cachexia. All patients and donors gave their informed voluntary consent to participate in the study.

Both the study and control groups were dominated by men (85% (68 out of 80 people) and 80.0% (32 out of 40 people), respectively). The median age was 60 years for the study group and 40 years for the control group. Histologically, squamous cell carcinoma was the most common type of cancer in the study group, affecting 65% of patients (52 out of 80), followed by adenocarcinoma in 33.8% (27 out of 80) of patients, and a rare type of pleomorphic tumor was found in one patient.

Staging was performed according to the international classification TNM 8th edition [19], and according to this, patients, taking into account the inclusion criteria (morphologically verified diagnosis of non-small-cell lung cancer, stage IIIB–IIIC of the disease), were distributed according to the protocol as follows: 48 people–stage IIIB and 32 people stage III.

The composition of lymphocyte populations in the peripheral blood from the healthy individuals, and the patients with lung cancer before any treatment, was determined using flow cytometry. Lymphocyte populations were evaluated in a direct immunofluorescence reaction using monoclonal antibodies to the main antigens of human lymphocytes conjugated with fluorochromes (CD3-FITC, CD3-PE, CD5-APC, CD8-PE, CD4-PerCP, CD19-PE-Cy7, CD56-PE-Cy7, CD16-FITC, CD45-APC-H7). The results were evaluated by flow cytometry using a 6-color BD FACS Canto II system (BD Biosciences, San Jose, CA, USA). Briefly, 1.5 mL of peripheral blood collected in EDTA was diluted in 0.4 mL flow cytometry buffer (PBS containing 10% *v*/*v* heat inactivated fetal calf serum, FCS). For each analysis, 100 µL of cell suspension was incubated with the fluorescent-labeled monoclonal antibodies for 20 min in the dark. After washing with flow cytometry buffer, erythrocytes were lysed by incubating with 2 mL of BD FACS™ Lysing solution (BD, Franklin Lakes, NJ, USA) (1:9 dilution in double distilled (dd) H_2_O, 349202-BD Biosciences) for 10 min in the dark at room temperature. After another washing step, the probes were analyzed in a FACSCanto II™ flow cytometer. Flow cytometry data were processed with Kaluza software, version 2.1.1.

**Figure 1:**
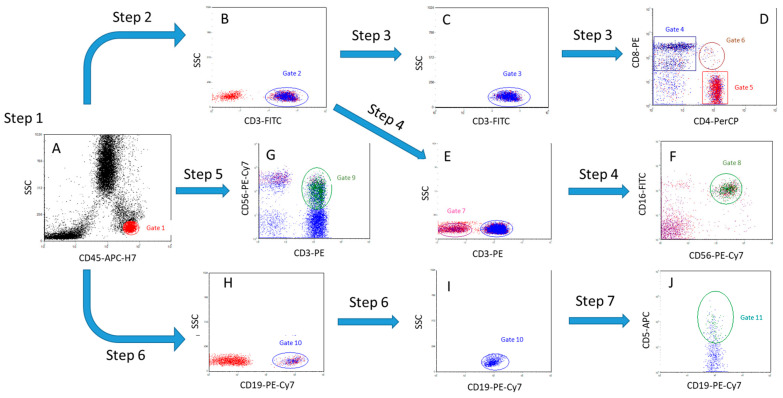
The gating algorithm. (**A**)—Step 1 (separation of a lymphocytes population), (**B**)—Step 2 (separation of T cells), (**C**,**D**)—Step 3 (separation of T cells into populations of CD4+ T helper cells, CD8+ cytotoxic T cells and double-positive CD4+CD8+ T cells), (**E**)—Step 4 (separation of CD3- cell population), (**F**)—Step 4 (separation of NK cells from CD3- cells by CD16+ and CD56+ markers), (**G**)—Step 5 (separation of NKT cell population), (**H**,**I**)—(separation of B cell population), (**J**)—Step 7 (separation of CD19 population+CD5+ B cells).

Gating strategy (Figure 1):

Step 1. Based on the expression of CD45 antigen and lateral light scattering (SSC), the lymphocyte gate (CD45++SSClow) was determined (A, gate 1, red);

Step 2. CD3+ cells (T cells) are isolated within the lymphocyte gate 1 (B, gate 2, blue);

Step 3. Among CD3+ cells (C, gate 3, blue), the number of CD3+CD8+ cytotoxic T cells (D, gate 4, blue), CD3+CD4+ T helper cells (D, gate 5, red,) and double-positive T cells CD4+CD8+ (D, gate 6, brown,) was determined;

Step 4. Within the population of CD3-negative lymphocytes (E, gate 7, pink,), the number of NK cells, CD3-CD16+CD56+, was estimated (F, gate 8, green);

Step 5. The number of NKT cells CD3+CD56+ was estimated within all lymphocytes (G, gate 9, green);

Step 6. The number of CD19+ B cells (H, gate 10, blue) was determined within all lymphocytes;

Step 7. Within CD19+ cells (I, gate 10, blue), the number of CD19+CD5+ B cells was estimated (J, gate 11, turquoise).

Statistical data processing was performed using the SPSS 2023 software. The results of the statistical analysis of the data on the studied quantitative characteristics were presented in the form of the median and interquartile range (median 25; 75), mean and standard error of the mean (SEM). To determine the reliability of differences between groups, the Mann–Whitney U-test was used, and to assess the dynamics of indicators, the Wilcoxon test was used. The analyzed indicators in the groups were compared using the *t*-test, and differences in values were considered significant at *p* < 0.05.

## 3. Results

The comparison of patient and donor indicators revealed a number of statistically significant differences. The blood samples from patients showed higher levels of white blood cells (9.1 × 10^3^/μL vs. 7.5 × 10^3^/μL *p* < 0.0001). However, the percentage of lymphocytes was significantly lower in patients (21.9% vs. 30.9% *p* < 0.0001). Despite this, the absolute number of lymphocytes did not differ significantly between the two groups due to the higher leukocyte count in patients.

**Figure 2:**
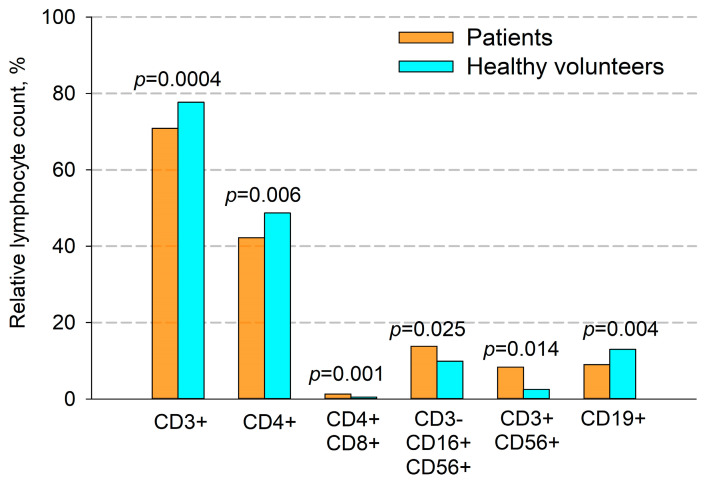
Comparison of the percentage of immune cells in patients with NSCLC and healthy volunteers. CD3+—T cells, CD4+—T helper cells, CD4+CD8+—double-positive T cells, CD3-CD16+CD56+—NK cells, CD3+CD56+—NKT cells and CD19+—B cells. Data are presented as mean ± SEM.

**Figure 3:**
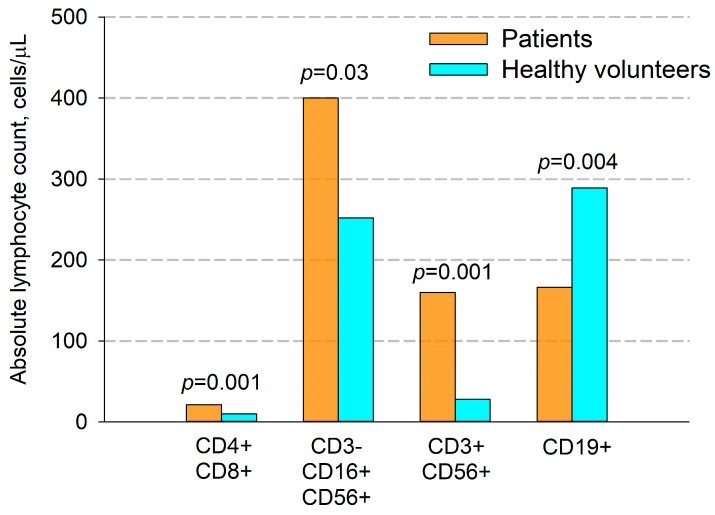
Comparison of absolute quantity (cells per μL) of immune cells in patients with NSCLC and healthy volunteers. CD4+CD8+—double-positive T cells, CD3-CD16+CD56+—NK cells, CD3+CD56+—NKT cells, CD19+—B cells. Data are presented as mean ± SEM.

It was found that in primary untreated patients with locally advanced unresectable NSCLC, there was a significant difference in the number of certain types of T cells compared to healthy volunteers (Figure 2 and Figure 3). Thus, the percentage of CD3+ (70.9% vs. 77.7%, *p* = 0.0004) and CD4+ (42.6% vs. 48.7%, *p* = 0.006) T cells was significantly lower in patients. The ratio of CD4+ to CD8+ T cells in patients was 1.96, while in healthy volunteers it was 2.07. The differences in absolute numbers turned out to be unreliable, but the trend continued. There was a significant increase in both the relative and absolute number of double-positive CD4+CD8+ T cells (1.25% vs. 0.5% and 21 cells/μL vs. 10 cells/μL, respectively, *p* = 0.001).

A significant increase in the percentage and the absolute amount of NK cells in patients were observed (CD3-CD16+CD56+, 13.8% vs. 9.9%, *p* = 0.025 and 399 cell/ μL vs. 252 cell/ μL, *p* = 0.03, respectively).

Additionally, in patients compared with donors, we found a significant increase in the number of NKT cells (CD3+CD56+) (Figure 2 and Figure 3), which was true for both their relative and absolute content. Thus, the average percentage of NKT cells in patients’ blood samples was 8.3%, and their absolute number per μL of blood was 160 cells, while in donors these figures were 2.5% and 28 cells per μL, respectively.

Also, in patients, compared to healthy volunteers, there was a significant decrease in both the percentage and absolute number of B cells (9.0% vs. 13.0%, and 166 cells/μL vs. 289 cells/μL, respectively, *p* = 0.004, Figure 2 and Figure 3).

**Figure 4:**
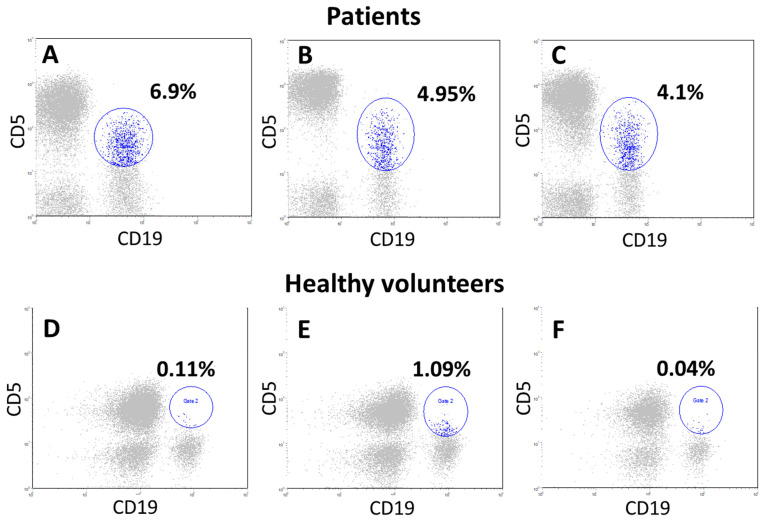
Cytograms of lymphocytes from primary patients with locally advanced NSCLC (**A**–**C**) and donors (**D**–**F**). First, lymphocytes (CD45++ SSC low cells, gate 1) were isolated from all blood cells by CD45 antigen expression. Further, CD19+ and CD5+ cells, as well as double-positive populations simultaneously expressing both CD19 and CD5 antigen (B1a cells), were evaluated within the lymphocytes (CD45++ SSC low cells), all cytograms show only lymphocytes (gray). Further, on all cytograms, along the abscissa axis, B cells (CD19+), along the ordinate axis of CD5+ cells, gate 2 (blue cells) these are CD19+CD5+ cells. In patient (**A**), the number of CD19+CD5+ cells was 4.1%, in patient (**B**)—6.9%, in patient (**C**)—4.95% within lymphocytes (CD45++ SSC low cells). Cytograms (**D**–**F**) are examples of blood lymphocyte samples from donors, where the CD5+B cell population was 0.11%,1.09%, and 0.4% of the lymphocytes, respectively (gate 2, blue cells).

Disorders affect not only quantitative indicators, but also the qualitative composition of individual components of the immune system. In a small group of NSCLC patients (12 individuals), we analyzed a specific subpopulation of B1a lymphocytes with the CD19+CD20+CD5+ immunophenotype. The number of these cells in 8 of the 12 patients (66.7% of cases) was significantly higher than in healthy volunteers, averaging 2.1% of all lymphocytes and 44.3% of B cells. However, statistical differences are unreliable due to the small sample (Figure 4).

**Figure 5:**
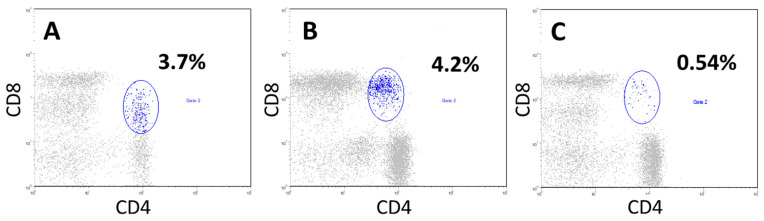
The presence of a pronounced proportion of double-positive CD4+CD8+ T cells in patients with locally advanced unresectable NSCLC at the onset of the disease in comparison with donors. In patients (cytograms (**A**,**B**)), CD4+CD8+ T cells among all blood lymphocytes accounted for 3.7% and 4.2%, respectively (gate 2, blue). Cytogram (**C**), a donor sample where CD4+CD8+T cells accounted for 0.54% of all lymphocytes (gate 2, blue).

In some patients, at the onset of the disease, we observed a significant increase in the proportion of CD4+CD8+ double-positive T cells (3.7%, with a threshold value of 1.0%), compared to the control group (Figure 5).

## 4. Discussion

The initial state of an oncological patient’s immune system, the ability of effector immune cells to activate, and the balance between effector and regulatory populations play a significant role in the success of specific therapies. Changes in the composition of these immunocompetent cells can serve as biomarkers for predicting a patient’s response to treatment [20,21,22].

This study is based on an analysis of our extensive clinical data comprising a cohort of 80 patients with newly diagnosed NSCLC who had not received anticancer treatment. The study demonstrates characteristic alterations in the subpopulations of peripheral blood lymphocytes, which may serve as predictors of response to therapy. In contrast to studies focused on a single cell type, this work employs a comprehensive approach, assessing the entire spectrum of circulating immune cells: T cells (including their main subpopulations), NK and NKT cells, and B lymphocytes. One of the innovative aspects of the study is the inclusion of a rare and poorly studied subgroup of B1a lymphocytes in the analysis, as well as the identification of changes in the number of double positive CD4+CD8+T cells.

The study showed that in patients with unresectable NSCLC, compared with donors, there are multidirectional changes in the quantitative composition of immune blood cells. In particular, a significant (*p* = 0.006) decrease in the number of CD3+CD4+ T cells was found. However, the CD4/CD8 ratio in patients and donors did not significantly differ (1.96 vs. 2.07, respectively).CD3+CD4+ cells belong to a population of T helper cells, effector cells that ensure the formation of an immunological synapse when interacting with antigen-presenting cells and promote the production of specific antibodies by B cells. A decrease in their number may be directly related to a decrease in the immune system’s response to a pathogen, including a tumor cell [23,24,25].

Interestingly, a higher CD4+/CD8+ ratio (i.e., a higher CD4+ cell count) in peripheral blood had a beneficial effect on the effectiveness of therapy, including radiation therapy in patients with rectal cancer [26]. This indicator may also be relevant for non-small cell lung cancer, but this requires additional research.

Cytolytic subpopulations of CD4+ T helper cells play a crucial role in the antitumor activity of the immune system. CD4+ T cells that have cytolytic functions against tumors have been well studied in melanoma [27]. Like most immune cells, the CD4+ T cell population is highly diverse, including small suppressor populations. The presence of these cells can negatively affect the course of the disease and response to treatment. Despite the decrease in the overall number of CD4+ T cells, there may also be an increased proportion of T regulatory cells among them, that was demonstrated in other types of cancer. In terms of predicting the outcome, a specific subpopulation of neoantigen-reactive T cells with an activated immunophenotype may be most significant for prognosis [28].

Noteworthy, of significant interest are double-positive CD4+CD8+ T cells, which are more characteristic of the thymus stage of T cell development. However, mature CD4+CD8+ T cells are also found in peripheral blood and peripheral lymphoid organs. This population is not homogenous, and their function (cytotoxic or suppressive) has not been clearly clarified yet [29]. Despite the marked diversity, double-positive CD4+CD8+ T cells usually account for less than 1% of the total number of T cells found in the blood of healthy people. It has been shown that the number of double-positive T cells in the blood of older people (for people over 65 years of age) is higher than in younger people [30].

These cells can stimulate inflammation due to their increased secretion of IL-10, interferon gamma (IFN-γ) and TGF-β. In the case of certain types of malignant neoplasms, such as Hodgkin’s lymphoma, melanoma, breast cancer and hepatocellular carcinoma, higher numbers of double-positive CD4+CD8+ T cells have been found. The increased prevalence of these cells in advanced stages of cancer, along with their cytotoxic properties and cytokine profile, suggest that they may be important in cancer progression. However, further research is needed to fully understand their role and potential as a target for therapy [31].

We found that in patients with locally advanced NSCLC, at the onset of the disease, there is a significantly higher number (both relative and absolute, *p* = 0.001) of double-positive CD4+CD8+ T cells among peripheral blood lymphocytes compared to healthy donors and it may be associated with the chronic inflammatory process associated with the disease. It should be noted that, more than 80% of cases of increased such cells have been reported in people under 60 years of age, which suggests that their increase is not related to age, but rather is a consequence of the development of tumor-associated inflammation.

Natural killer (NK) and natural killer T (NKT) cells are two important components of the innate immune system. These two cell types have similar phenotypic characteristics and play a role in the antitumor immune response. There is evidence regarding the clinical significance of the number of NK cells at the onset of NSCLC. Favorable clinical outcomes have been associated with a high number of active NK cells, as shown in a study by Mazzaschi et al. [32]. Additionally, in experimental study [33], it was found that at the early stages of tumor development (day 10), there was a higher absolute number of NK cells in the blood of mice with E0771 transplanted tumor compared to mice without a tumor. However, as the tumor size increased and progressed, the absolute number of NK cells decreased in the blood. The combination of a high number of circulating NK cells with a low ratio of PD-1 to CD8+ among tumor-infiltrating lymphocytes, characterized the group with the best prognosis and the longest progression-free survival [33].

NKT cells show the characteristics of innate as well as adaptive immune cells. NKT cell activation leads to rapid production of inflammatory cytokines and modulates the function of several effectors and regulatory immune cells both in mice and humans [34]. According to the nature of the activating ligand, NKT cells are classified into two groups: type-I and type-II NKT cells. Individual studies have shown that the number of NKT cells in the peripheral blood of patients with multiple myeloma and breast cancer is reduced compared to healthy donors. There is also a reduced number of circulating NKT cells in patients with squamous cell carcinoma of the head and neck, which is associated with poorer patient survival [35,36].

In locally advanced small-cell lung cancer, on the contrary, an increase in the number of circulating NKT cells was observed [37]. In our study, patients exhibited a relative increase in the total number of NKT cells with the CD3+CD56+ immunophenotype, as well as an increase in both relative and absolute numbers of NK cells (CD3-CD16+CD56+) as compared to healthy volunteers. These NKT cells findings are consistent with the data obtained for ovarian cancer and breast cancer from a study by Tabakov D.V. et al. [38], as well as for epithelial cancers from earlier studies [37]. With regard to NK cells, similar data have been obtained by Shubina I. et al. in ovarian cancer [39]. However, this study included not only patients with primary disease, but also those who underwent chemotherapy.

It is possible that the role of these cells should be considered only in the context of individual types of cancer and the stage of the disease.

It is important to note that the populations of NKT and NK cells were considered as a potential candidate for cell therapy of NSCLC. However, unfortunately, the benefit in terms of the duration of the recurrence-free survival has not been significant, and more research is needed to understand the reason for this insignificance and to develop more effective cell therapy strategies [40,41,42,43].

Mature B cells, which are a key component of the adaptive immune response, play a crucial role in the antitumor response as well. These cells secrete pathogen-neutralizing antibodies and pro-inflammatory cytokines or turn into memory B cells, providing long-term protection against pathogens. These cells can also function as alternative antigen-presenting cells [44,45]. Our study found that in patients with locally advanced NSCLC the total B-cell count in peripheral blood was decreased. This may indicate a failure of humoral immune response. Interestingly, along with a decrease in the total B cell count among peripheral blood lymphocytes, in some patients we found a significantly higher count of a specific population of B cells that express the CD5 antigen (CD19+CD20+CD5+ B cells). This cell subpopulation is known as B1a lymphocytes secreting predominantly IgM, a type of natural antibody that plays a key role in apoptosis of tumor cell [46,47].

Among B1a lymphocytes, there is a special subpopulation of cells (B10 cells) that secrete IL-10, a cytokine with immunosuppressive properties. Experimental models have shown that this subpopulation promotes tumor growth, particularly in melanoma [48,49]. Normally, the B1a population makes up less than 10% of peripheral B-cells. However, in 23% of gastric cancer patients, was found a significant increase in B1a cells, exceeding the normal level by two times but in some patients, their count exceeded 40%, as shown by us earlier [50,51]. In this study we showed that the B1a lymphocytes count in the peripheral blood of patients with locally advanced NSCLC at the onset of the disease was significantly higher than in healthy volunteers. However, due to the small sample size, the differences were not statistically significant as only 12 patients were analyzed from the entire group. Further studies are needed to assess the clinical significance of this subpopulation of B cells.

## 5. Conclusions

Thus, patients with NSCLC show quantitative and qualitative changes in the levels of certain populations of immune cells. The revealed multidirectional changes in immune cells in patients with locally advanced NSCLC (stages IIIB–IIIC) confirm the presence of signs of secondary immunodeficiency in this group of patients. The clinical significance of these changes needs further investigation, in particular individual functional tests, assessment of T-regulatory cells and humoral immunity (cytokines and complement system), but it is already evident that in patients with primary NSCLC, it is necessary to assess immunological parameters in blood and consider the possibilities of immunotherapeutic support.

## Figures and Tables

**Table 1:** Characteristics of patients.

Criteria	Indicator
Age	39–82 years (the median -60 years)
Gender	male -68 pts female -12 pts
ECOG status	1 (all patient)
Histological subtype of the tumor	SCC—52 pts AC—27 pts pleomorphic—1 pts
Stage of the disease	IIIB—48 pts IIIC—32 pts
Chemotherapy	SCC-paclitaxel 175 mg/m^2^ on Day 1 + carboplatin AUC 5 on Day 1; cycle 21 days AC-pemetrexed 500 mg/m^2^ on Day 1 + cisplatin 75 mg/m^2^ on Day 1; cycle 21 days
Smoking experience	10–60 years
Concomitant pathology	Hypertension—30 pts History of hepatitis B—3 pts Gastric ulcer in remission—5 pts Chronic heart failure—23 pts Diabetes mellitus—11 pts Atherosclerosis of the aorta and blood vessels—8 pts
Follow-up (median observation for February 2025)	19.2 months

## Data Availability

The datasets used and/or analyzed during the current study are available from the corresponding author upon reasonable request.

## Abbreviations

The following abbreviations are used in this manuscript:

AC	Adenocarcinoma
CCRT	Concurrent chemo-radiotherapy
ICIs	Immune checkpoint inhibitors
IFN-γ	Interferon gamma
NK	Natural killer cells
NKT	Natural killer T cells
NSCLC	Non-small-cell lung carcinoma
SCC	Squamous cell carcinoma
SSC	Side Scatter
TGF-β	Transforming growth factor beta
TNM	Tumor, lymph node, metastasis
